# *ABCG2* Polymorphisms and Predictive Fluoroquinolone Phototoxicity in Nondomestic Felids

**DOI:** 10.3390/genes13122178

**Published:** 2022-11-22

**Authors:** Alexandria E. Gochenauer, Dayna L. Dreger, Brian W. Davis, Shawna Cook, Katie E. Barber, Kari J. Ekenstedt

**Affiliations:** 1 Veterinary Hospital, College of Veterinary Medicine, Purdue University, 625 Harrison Street, West Lafayette, IN 47907, USA; 2 Department of Basic Medical Sciences, College of Veterinary Medicine, Purdue University, 625 Harrison Street, West Lafayette, IN 47907, USA; 3 Department of Veterinary Integrative Biosciences, College of Veterinary Medicine, Texas A&M University, 660 Raymond Stotzer Parkway, College Station, TX 77843, USA; 4 Department of Pharmacy Practice, School of Pharmacy, University of Mississippi, 2500 N State Street, Jackson, MS 39216, USA

**Keywords:** enrofloxacin, missense variant, retina, lion, tiger, cheetah, leopard, cougar, *ABCG2* gene

## Abstract

Fluoroquinolones are a widely used class of chemotherapeutics within veterinary medicine, prized for their broad-spectrum bactericidal activity. These drugs present a known risk of retinal phototoxicity in domestic cats (*Felis catus*); therefore, using lower doses and alternative antibiotic classes is encouraged in this species. This adverse drug effect of fluoroquinolones, and enrofloxacin specifically, has been determined to be species-specific in domestic felids. Four feline-specific missense variants in *ABCG2* result in four amino acid changes (E159M, S279L, H283Q, and T644I) that are unique to the domestic cat compared with multiple other nonfeline mammalian species. These changes alter the ABCG2 protein involved with the cellular transmembrane transport of drugs, including fluoroquinolones, making the protein functionally defective in domestic cats. The predisposition to fluoroquinolone-mediated phototoxicity in nondomestic felids was explored in this study. At least eight nondomestic felids share the four *ABCG2* missense variants with domestic cats, and eleven other felids shared at least three of the four domestic cat variants. Taken together, these results suggest the genetic potential for nondomestic felids to also experience fluoroquinolone-induced retinal phototoxicity; therefore, cautions similar to those for domestic cats should be followed for these drugs in the entire feline taxon.

## 1. Introduction

In 2005, the World Health Organization designated fluoroquinolones, a class of medications utilized in both human and veterinary medicine, as a critically important, highest priority antimicrobial group [1]. Quinolones are still listed as a critically important antimicrobial today. Enrofloxacin is a fluoroquinolone used in veterinary patients for a variety of susceptible infections [2], and the fluoroquinolone class of drugs is often utilized as a broad-spectrum antibiotic while awaiting a susceptibility panel or in cases where other antibiotics are ineffective due to resistance [3]. Fluoroquinolones have intense bactericidal activity and function by inhibiting gyrase (a type-II topoisomerase) and topoisomerase IV, which interferes with the supercoiling of bacterial chromosomal material [4]. The broad-spectrum activity of these antibiotics is due to the primary targeting and inhibiting of gyrase in gram-negative bacteria and topoisomerase IV in most gram-positive organisms. Due to differences in sequence and structure, the topoisomerase enzymes of eukaryotic cells are unaffected.

Fluoroquinolones carry a risk of ocular phototoxicity in domestic cats (*F. catus*); therefore, using lower doses and alternative antibiotic classes is encouraged in this species [2]. This recommendation is based on earlier work demonstrating that domestic cats receiving parenteral enrofloxacin in rare cases developed acute blindness from enrofloxacin-induced retinal degeneration [5]. Although the mechanism of the ocular phototoxicity was not then fully understood, it became clear that increasing age in the domestic cat patient and overdosing beyond the recommended dosage played a role in the phototoxicity that occurred [6]. Further work demonstrated that enrofloxacin at a level 10 times higher than the normal dose is acutely toxic to the outer retina in healthy cats [7]. Fluoroquinolones can also induce phototoxic skin reactions, typically appearing first in white-haired, nonpigmented, or hairless areas, and affecting even black-coated animals if the light is bright enough or with long enough exposure [8].

These phototoxic reactions are now explained by a better understanding of the cellular and molecular interactions of this group of drugs. Fluoroquinolones and many other drugs are substrates for the membrane-associated protein ATP Binding Cassette Subfamily G Member 2, encoded by the *ABCG2* gene, which is involved in the transmembrane efflux of drugs across cell membranes [9,10]. In the retina specifically, ABCG2 is located on the luminal membrane of retinal capillary endothelial cells, where this protein functions to actively transport its substrates from the endothelial cell back into the capillary lumen [11].

*ABCG2* is part of the larger *ATP Binding Cassette* (*ABC*) gene family, where it is one of only four members (*ABCB1*, *ABCG2*, *ABCC1*, and *ABCC2*) implicated in transporting commonly used drugs across cell membranes in veterinary patients [12]. ABC transporters use energy from ATP hydrolysis to export not only exogenous drugs but also exogenous metabolic products such as lipids and sterols [13]. A four-base pair (bp) deletion in *ABCB1* (previously *MDR1*), observed commonly in herding breeds of domestic dogs (*C. lupus familiaris*) [14], is well-known for causing the toxicity associated with a number of medications, including sedatives, chemotherapies, dexamethasone, and fluoroquinolones [15], but most famously with anthelminthics such as ivermectin. The *ABCB1* deletion mutation results in a loss of functional ABCB1 efflux transporter in affected dogs [16] and was recently explored in a small cohort of nondomestic canine species (maned wolf, *Chrysocyon brachyurus*; gray wolf, *Canis lupus*; red wolf, *C. rufus*; coyote, *C. latrans*; dingo, *C. lupus dingo*; New Guinea singing dog, *C. lupus dingo*; arctic fox, *Vulpes lagopus*; and fennec fox, *V. zerda*) [16]. These exotic canids were tested for the dog *ABCB1* variant and shown to be entirely wild-type [16], suggesting that these species are protected from the toxicities observed in herding dogs. This naturally leads to the question of if *ABCG2* in exotic felids has similar sequence changes and functional alterations as in the domestic cat, which would have implications for the antibiotic selection and dosing.

Certain *ABCG2* polymorphisms have already been demonstrated to result in reduced transport activity with or without decreased protein expression in humans [9]. These patients have an increased systemic exposure to substrate drugs compared to patients with wild-type *ABCG2*, increasing the likelihood of adverse drug reactions. Similar to ABCB1, ABCG2 plays a major role in drug-dependent pharmacokinetics [12], and its involvement in drug transport also helps protect the host from potential toxicities by restricting the access of drugs to sensitive tissues [11]. For example, since ABCG2 is expressed at the inner blood-retina barrier, and since fluoroquinolones are substrates for ABCG2, the distribution of fluoroquinolones to the retinal tissue is normally restricted [11]. The fluoroquinolone-induced retinal phototoxicity experienced by domestic cats, particularly from enrofloxacin, was determined to be species-specific [5,6,7]. Four ABCG2 amino acid changes (E159M, S279L, H283Q, and T644I) were specific to the domestic cat, when compared with 10 other mammalian species [11]. The phototoxic process in domestic cats was confirmed to be the result of a deficiency in the ABCG2 drug efflux of fluoroquinolones [11]. However, little is known about the predisposition to fluoroquinolone-induced phototoxicity in nondomestic felids. While enrofloxacin has been used previously in lions (*Panthera leo*) and tigers (*P. tigris*) [17], dosing has mostly been at the lower recommendations of 5 mg/kg once daily [18] out of concern for toxic reactions. Higher doses, if nontoxic, would increase efficacy; therefore, the goal of this study was to sequence over the same four *ABCG2* missense variants from *F. catus* in 19 nondomestic felid species to determine the possibility of these species experiencing phototoxic reactions similar to the domestic cat. It was hypothesized that if these same amino acid changes were present, it would provide clues about genetic potential for fluoroquinolone-induced retinal phototoxicity across the felid taxon.

## 2. Materials and Methods

### 2.1. Animals, Ethics Statement, and Collection of DNA

All animals in this study were from public and private North American zoological facilities, and samples were opportunistic as residual blood samples originally drawn for other purposes and banked as EDTA whole blood (IACUC approvals not required). Due to the opportunistic sampling, no phenotypes (e.g., medical history) were available other than species identification. Nineteen feline species were sampled (*n* = 32) (Table 1), typically with one individual per species. However, three species (cougars (*P. concolor*), lions, and tigers) were represented by four, two, and ten samples, respectively. Genomic DNA was extracted (Gentra Puregene kit, Qiagen, Hilden, Germany) per manufacturer’s instructions and stored at −80 °C.

### 2.2. Targeted Genotyping

The domestic cat reference genome (Felis_catus_9.0) was accessed via Ensembl (https://useast.ensembl.org/Felis_catus, accessed 21 September 2022). Sequence flanking each of the four previously published *F. catus ABCG2* missense variants (E159M, S279L, H283Q, and T644I) [11] was used to design primers such that, to the extent possible, each primer was located within exon boundaries in order to increase their successful utility for multiple species. These four variants will be referred to by their original domestic cat nomenclature (E159M, S279L, H283Q, and T644I) throughout this study to provide cohesion. Primers were designed in Primer3 (http://bioinfo.ut.ee/primer3-0.4.0/, accessed 21 September 2022) and manufactured by IDT (Integrated DNA Technologies, Coralville, IA, USA) (Table 2). All primer pairs were optimized and verified using pooled domestic cat DNA obtained from surplus hospital samples (Purdue University Veterinary Hospital, West Lafayette, IN, USA; client consent obtained; no IACUC required) to confirm accuracy and specificity of each primer pair.

**Figure A1:**
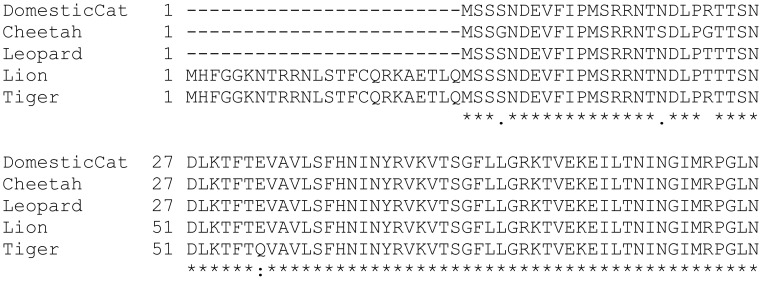

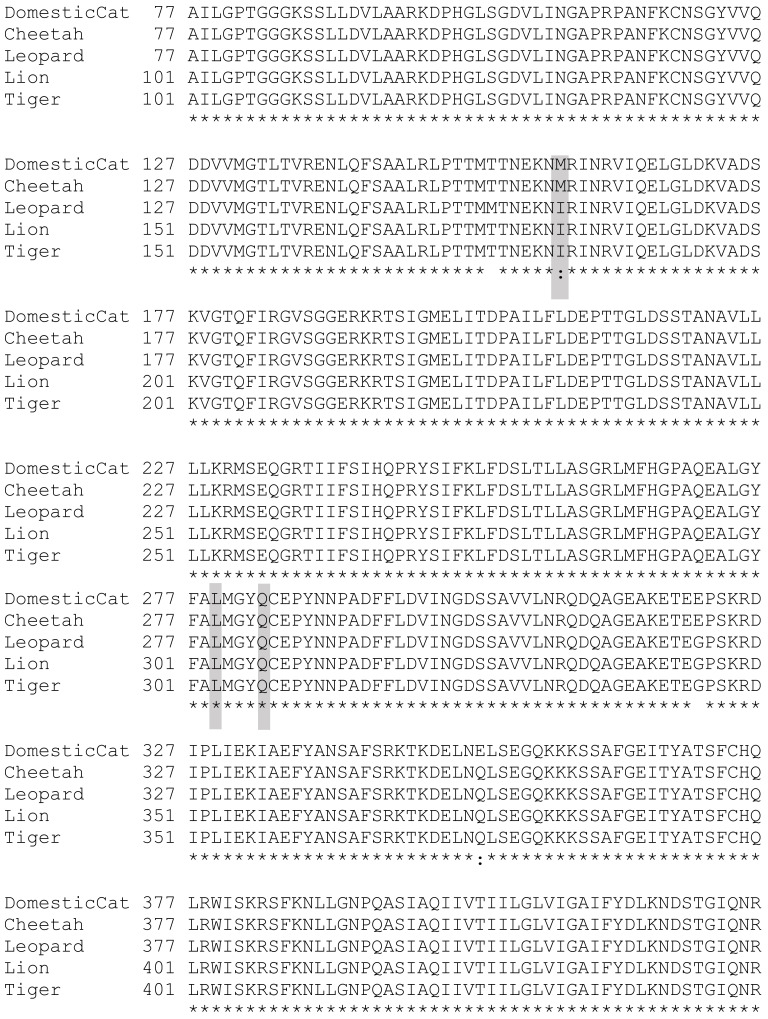

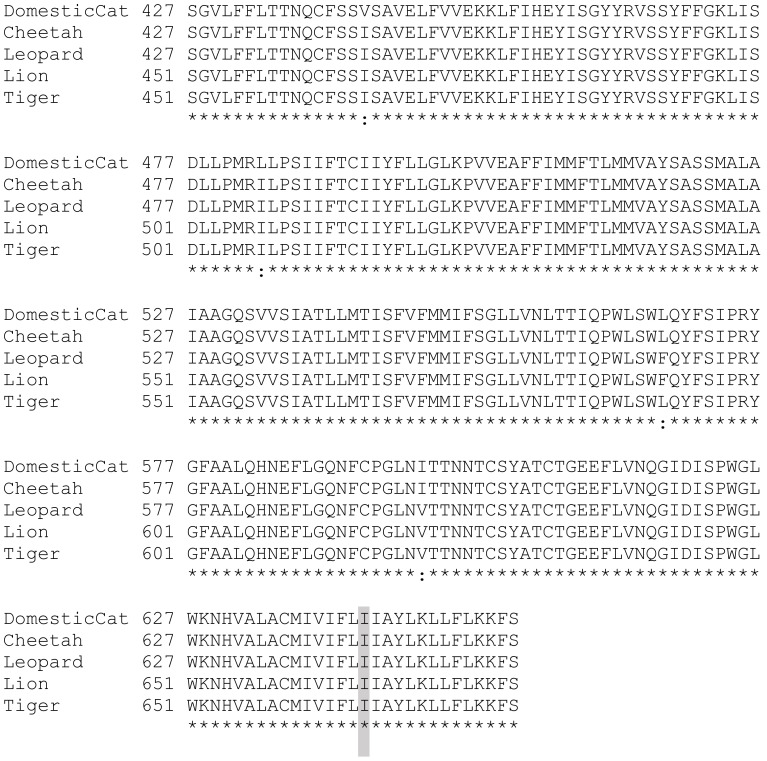
*ABCG2* amino acid alignment for domestic cat, cheetah, leopard, lion, and tiger. * All data were accessed on 21 September 2022 via NCBI. Genome assembly builds accessed are FelCat9.0 (domestic cat, XP_023108715.2), Aci_jub_2 (cheetah, XP_026916402.1), PanPar1.0 (leopard, XP_019320292.1), P.leo_Ple1_pat1.1 (lion, XP_042791867.1), and P.tigris_Pti1_mat1.1 (tiger, XP_042840143.1). The four domestic cat missense amino acid changes studied in this paper are highlighted in grey.

Polymerase chain reaction (PCR) and Sanger sequencing were used to generate genotypes for each animal at each of the four variants. PCR products from genomic DNA were amplified using KOD Hot Start DNA Polymerase (Sigma-Aldrich, St. Louis, MO, USA) in a 57–55 °C step down protocol, verified on 1% agarose gels, purified, and then Sanger sequenced (Eurofins Genomics, Louisville, KY, USA). Sequence chromatograms were analyzed in Sequencher software (v5.1, GeneCodes, Ann Arbor, MI, USA), together with the domestic feline reference sequence (FelCat 9.0), and each sequence was manually visualized to determine the genotype. Amino acid alignments for the ABCG2 protein from a representative subset of available draft genome assemblies (domestic cat, FelCat 9.0; cheetah, Aci_jub_2; leopard, PanPar 1.0; lion, P.Leo_Ple1_pat1.1; and tiger, P.tigris_Pti1_mat1.1) were built (Appendix A, Figure A1) using T-Coffee [19,20]. Assemblies were also publicly available for eight additional species (Asian leopard cat, *P. bengalensis*; black-footed cat, *F. nigripes*; caracal, *C. caracal*; cougar, *P. concolor*; fishing cat, *P. viverrinus*; jaguarundi, *H. yagouaroundi;* jungle cat, *F. chaus*; and snow leopard, *P. uncia*). *ABCG2* sequences from publicly available whole genome sequences for the 12 available nondomestic feline species were compared to those generated in this work.

## 3. Results

All 32 samples were successfully sequenced over three of the four missense variants (E159M, S279L, and T664I) (Table 3). However, only 27 samples were successfully sequenced for the remaining missense variant, H283Q. For the first variant, E159M, out of the 19 species tested in this study, five had a different variant from the domestic cat, while 14 matched the domestic cat. All 19 species were identical to the domestic cat at the second variant, S279L. For all the species (*n* = 14) with successful amplification over the third variant (H283Q), their sequence also matched that of the domestic cat. For the fourth variant, T664I, 18 species matched that of domestic cat, with margay coding for a threonine residue (Thr, T), which matches the residue found in other nonfeline species (e.g., human, chimpanzee, macaque, cattle, sheep, goat, horse, mouse, rat, and dog). The amino acid alignment generated from a representative subset of public genome assemblies (Figure A1), which includes the domestic cat, leopard, lion, tiger, and cheetah, confirmed all findings for those species. Indeed, the comparison of all publicly available genome assemblies (*n* = 12, Table A1) confirmed all the sequencing results in this study for those species. Additionally, these genome assemblies indicated that the Asian leopard cat, caracal, and fishing cat likely also share the same sequence with the domestic cat at the H283Q variant, although these three species were not successfully sequenced over this locus in this work.

In total, at least eight feline species (black-footed cat, cheetah, clouded leopard, cougar, Asian golden cat, jaguarundi, jungle cat, and marbled cat) shared the exact same four *ABCG2* missense variants of the domestic cat. When the sequence from available genomes is included, this number rises to 11 with the addition of the Asian leopard cat, caracal, and fishing cat at the H283Q locus. An additional 11 felids sequenced in this study (Asian leopard cat, caracal, fishing cat, leopard, lion, margay, Pallas’s cat, serval, snow leopard, tiger, and tigrina) shared at least three of the four domestic cat variants. When multiple samples were available for a species (cougar, lion, tiger), the same genotyping results were observed for all individuals within a species.

## 4. Discussion

This pharmacogenetic study provides insights into the potential behavior of fluoroquinolones in nondomestic feline species. Given the utility of this group of antibiotics, particularly enrofloxacin, many scenarios are likely where their use would benefit nondomestic felids, if they are not contraindicated. One recent retrospective study investigated the possibility of enrofloxacin-associated retinal phototoxicity in lions and tigers by studying the postmortem thickness of the outer nuclear retina. These felids were administered enrofloxacin orally at the recommended oral administration dose for domestic cats (5 mg/kg by mouth once daily) for 14 days; postmortem, no thinning of the outer nuclear retinal layer was found in any patients [17]. This finding is not surprising, considering the animals received the lower, domestic cat dosing. The current formulary dosing recommendations for enrofloxacin are published for leopards (8 mg/kg once daily), African lions (1.1 mg/kg once daily), and a general dose for large felids (5 mg/kg once daily) [18]. The question remains whether or not nondomestic cat species would experience retinal phototoxicity from this group of drugs, which this study demonstrates is likely.

Previous work identified four nonconserved feline-specific missense variants and suggested that (1) these amino acid changes affect the protein trafficking ability of ABCG2, (2) *F. catus* ABCG2 transport function of key substrates is impaired, and (3) *F. catus* ABCG2 may have changes in substrate specificity [11]. They further demonstrated that UV light exposure combined with increased enrofloxacin concentrations led to increased phototoxicity. The UV light exposure is key because fluoroquinolones generate reactive oxygen species when thus exposed; the reactive oxygen species then attach to the retinal cellular lipid membranes and create tissue damage, which leads to the stimulation of apoptosis [11]. This phototoxic effect can occur in both natural and artificial light [11]. Indeed, quinolones were a known phototoxin; as early as 1993, they were reported to cause phototoxic reactions in albino mice after a single dose and in combination with ultraviolet A light [8].

All four *F. catus* missense variants are in highly conserved areas of the ABCG2 protein [11]. However, it is not clear which one, or which combination, is ultimately responsible for ABCG2’s decreased transport efficacy. In this study, the four domestic cat variants were examined in 19 nondomestic feline species. Moving sequentially through the *F. catus* ABCG2 protein, the first missense SNP variant (E159M) is a glutamic acid in 10 other mammals, including humans, mice, and dogs; domestic cats have a methionine residue at this position [11]. Fourteen of the nondomestic felids sequenced in this study also had methionine, resulting from the same missense variant. Five of the feline species, the leopard, lion, serval, snow leopard, and tiger, have sequence coding for yet another amino acid, isoleucine. Both methionine and isoleucine are nonpolar and hydrophobic, although isoleucine lacks the sulfur found in methionine. It is possible that isoleucine, like methionine, changes the ABCG2 protein structure, since both are chemically different from the acidic residue, glutamic acid, seen in other mammals. The E159M variant, and the E159I variant described in this study, occur in an ATP binding domain of the ABCG2 protein; an earlier study determined that a Q141K amino acid change in the same domain in humans leads to a substantial decrease in ABCG2 transport efficacy [21]. This further supports the implication that feline variants at this locus are related to fluoroquinolone-induced retinal phototoxicity.

The next *F. catus* missense SNP (S279L) [11], a leucine in the domestic cat, is a serine in seven mammals, including humans, mice, and dogs. All 19 nondomestic feline species sequenced in this study possessed a leucine residue at this locus. However, this particular amino acid residue is not as highly conserved overall; sheep and goats have an aspartic acid for this residue, and cattle have an alanine. The serine, which is polar and uncharged, is different chemically from the leucine and alanine, which are nonpolar and hydrophobic, and the acidic aspartic acid. Based on the many differences among mammalian species at this site, it seems less likely that the feline leucine (S279L) is pivotal in altering drug transport ability and, therefore, enrofloxacin sensitivity.

The next *F. catus* missense SNP variant (H283Q) is a histidine residue in 10 other mammals, including humans, mice, and dogs, whereas the domestic cat possesses a glutamine residue at this locus [11]. Samples from 14 species sequenced over this locus in this study all had the identical missense variant coding for a glutamine residue. Samples from five species (Asian leopard cat, caracal, fishing cat, Pallas’s cat, and tigrina) failed to amplify over this region; however, the available genome sequence for the Asian leopard cat, caracal, and fishing cat (Fcat_Pben_1.1_paternal_pri, CarCar 1.0, and UM_Priviv 1.0, respectively) also showed a match to the domestic cat at this locus, bringing the total to 17. The failed sequencing in this study is most likely due to the sample condition and primer design, and this locus should eventually be confirmed in future work. Histidine is a basic amino acid with a bulky ring structure, whereas glutamine is polar, uncharged, and lacks a ring structure, differences that could potentially impact ABCG2 protein function.

The fourth *F. catus* missense SNP (T664I) is highly conserved, with the threonine residue again being present in 10 other mammals. The domestic cat possesses an isoleucine at this locus. Of the 19 species studied here, 18 also possessed an isoleucine residue, identical to the domestic cat. The only outlier, the margay, did not have the variant, and its sequence codes for threonine, as seen in other mammals. Given that the threonine residue is polar but uncharged, while the isoleucine is nonpolar and hydrophobic, this fourth missense variant also potentially impacts protein function.

Since it remains unknown which of the four, or which combination of, amino acid changes led to the decrease in transporter efficacy, each of the tested feline species should be considered in combination across all four loci. A total of eight felids (black-footed cat, cheetah, clouded leopard, cougar, Asian golden cat, jaguarundi, jungle cat, and marbled cat) share the exact same four domestic cat *ABCG2* missense variants; therefore, the ABCG2 protein is expected to behave similarly in these species as in the domestic cat (i.e., poor transporter function). A further 11 felids (Asian leopard cat, caracal, fishing cat, leopard, lion, margay, Pallas’s cat, serval, snow leopard, tiger, and tigrina) sequenced in this study shared at least three of the four domestic cat variants. For five of these eleven, the differing variant was the H283Q locus, where data are missing due to the difficulty sequencing over the variant; of these five, three, the Asian leopard cat, caracal, and fishing cat, did match the domestic cat at this locus, based on publicly available reference genome assemblies. Given these findings, it seems very likely that the genetic potential exists for most, if not all, of the felids tested herein to experience fluoroquinolone-induced retinal phototoxicity. Certainly, given the limitation of the very small sample size for most of the species tested, future research efforts should confirm these findings in more individuals. Likewise, future work would benefit significantly from the addition of the medical history and other phenotype data for these animals, in combination with their genotypes for these variants.

Although further work is needed to understand the exact contributions of each of the missense variants to the overall efficacy of the ABCG2 protein, it is clear that careful consideration must be applied before using the fluoroquinolone group of antibiotics in nondomestic felids. Given the broadly shared missense changes across the included species, it would also be prudent to apply such consideration to any felid, even those not tested in this study. As in domestic cats, if a nondomestic felid also has renal or liver disease, such that fluoroquinolone elimination would be impaired [10], extra precautions must be taken. It is also probably best practice for any felid treated with fluoroquinolones to have limited exposure to natural and artificial light. Interestingly, newer generation fluoroquinolones, such as pradofloxacin and marbofloxacin, have proven to be safer alternatives in the domestic cat. Studies have shown that marbofloxacin at 10 times the highest labeled dose did not cause retinal lesions when administered to cats [22]. Pradofloxacin has also been shown to have better ocular safety than enrofloxacin, and a pharmacokinetic and pharmacodynamic study suggests that it should be the drug of choice when fluoroquinolones are indicated [23]. Another option, orbifloxacin, carries a precautionary statement about its use in cats due to retinal toxicity [24]; however, according to the United States Pharmacopeia, high doses of orbifloxacin produced minimal photoreceptor degeneration in cats [25]. Therefore, while the findings of this study suggest that enrofloxacin should only be administered to nondomestic felids at low doses, as in domestic cats, or avoided altogether, it is possible that the newer fluoroquinolones may be a better option for treatment.

## 5. Conclusions

The results of this study support the genetic potential for nondomestic felids to experience fluoroquinolone-induced retinal phototoxicity, and extra care should be taken when determining antibiotic treatment plans for this taxon.

## Figures and Tables

**Table 1:** Nondomestic felid species genotyped for *ABCG2* variants.

Species	Animals
Asian leopard cat (*Prionailurus bengalensis*)	1
Black-footed cat (*Felis nigripes*)	1
Caracal (*Caracal caracal*)	1
Cheetah (*Acinonyx jubatus*)	1
Clouded leopard (*Neofelis nebulosi*)	1
Cougar (*Puma concolor*)	4
Fishing cat (*Prionailurus viverrinus*)	1
Asian golden cat (*Catopuma temminckii*)	1
Jaguarundi (*Herpailurus yagouaroundi*)	1
Jungle cat *(Felis chaus*)	1
Leopard (*Panthera pardus*)	1
Lion (*Panthera leo*)	2
Marbled cat (*Pardofelis marmorata*)	1
Margay (*Leopardus wiedii*)	1
Pallas’s cat (*Otocolobus manul*)	1
Serval (*Leptailurus serval*)	1
Snow leopard (*Panthera uncia*)	1
Tiger (*Panthera tigris*)	10
Tigrina (Oncilla) (*Leopardus tigrinus*)	1
**Total**	**32**

**Table 2:** Primer Pair Sequences Used to Amplify *ABCG2* Variants.

Variant	Primer	Sequence	Product Size (BP)
E159M	SNP1F	ATGTCGTGATGGGCACTCTG	156
	SNP1R	CACATTACCTTGGAATCCGCC	
S279L	SNP2F	TCTGAACAGGGACGAACAATCA	221
	SNP2R	TCCTATAGACCCCTTTTACTCTCT	
H283Q	SNP3F	TCACCTCACTCACCCCTGTA	190
	SNP3R	TCATACCTTCACCTGCCTGG	
T664I	SNP4F	TGTTGAATTTCAGATGTACTGGCG	193
	SNP4R	TGTGCACATAAATCATGTAAATTCAAG	

**Table 3:** Sequencing results for the four domestic cat *ABCG2* missense variants in nondomestic felids. One individual per species unless otherwise indicated.

Species	E159M AA ^a^ = (ATG)	S279L AA = (TTA)	H283Q AA = (CAG)	T664I AA = (ATA)
Asian leopard cat	X ^b^	X	# ^c^	X
Black-footed cat	X	X	X	X
Caracal	X	X	# ^c^	X
Cheetah	X	X	X	X
Clouded leopard	X	X	X	X
Cougar (*n* = 4)	X	X	X	X
Fishing cat	X	X	# ^c^	X
Asian golden cat	X	X	X	X
Jaguarundi	X	X	X	X
Jungle cat	X	X	X	X
Leopard	ATA (=I)	X	X	X
Lion (*n* = 2)	ATA (=I)	X	X	X
Marbled cat	X	X	X	X
Margay	X	X	X	ACA (=T)
Pallas’s cat	X	X	? ^d^	X
Serval	ATA (=I)	X	X	X
Snow leopard	ATA (=I)	X	X	X
Tiger (*n* = 10)	ATA (=I)	X	X	X
Tigrina	X	X	? ^d^	X

^a^ AA = amino acid codon, listed as the codon found in domestic cat. ^b^ X = indicates the species listed matches the sequence found in domestic cat and would therefore have the same AA at this location. When species does not match domestic cat, the sequence for that codon, and its coded AA (single letter abbreviation), are provided. ^c^ # = no sequence was successfully generated over the missense variant; however, publicly available genome sequence indicates a match with domestic cat. ^d^ ? = no sequence was successfully generated over the missense variant, and no reference genome exists to date.

## Data Availability

The data are contained within the article and appendix.

## Appendix A

**Table A1:** Non-domestic felids with publicly available whole genome builds.

Species	Scientific Name	Assembly Number	Assembly Name	Assembly Level
Asian leopard cat	*P. bengalensis*	GCF_016509475.1	Fcat_Pben_1.1_paternal_pri	Chromosome
Black-footed cat	*F. nigripes*	GCA_004023925.1	FelNig_v1_BIUU	Scaffold
Caracal	*C. caracal*	GCA_016801355.1	CarCar1.0	Scaffold
Cheetah	*A. jubatus*	GCF_003709585.1	Aci_jub_2	Scaffold
Cougar	*P. concolor*	GCF_003327715.1	PumCon1.0	Scaffold
Fishing cat	*P. viverrinus*	GCF_022837055.1	UM_Priviv_1.0	Chromosome
Jaguarundi	*P. yagouaroundi*	GCF_014898765.1	PumYag	Scaffold
Jungle cat	*F. chaus*	GCA_019924945.1	FelChav1.0	Chromosome
Leopard	*P. pardus*	GCF_001857705.1	PanPar1.0	Scaffold
Lion	*P. leo*	GCA_018350215.1	P.leo_Ple1_pat1.1	Chromosome
Snow leopard	*P. uncia*	GCF_023721935.1	Puncia_PCG_1.0	Chromosome
Tiger	*P. tigris*	GCF_018350195.1	P.tigris_Pti1_mat1.1	Chromosome

All assemblies accessed September 2022.
